# Prevalence of Clostridium Difficile Infection (CDI) among Inflammatory Bowel Disease (IBD) Patients in Comparison to Non-IBD Patients in King Abdulaziz Medical City in Jeddah

**DOI:** 10.1155/2023/9958104

**Published:** 2023-10-13

**Authors:** Ghassan Abdulrahman Sukkar, Syed Sameer Aga, Abdulrahman Hamid Alsamadani, Faisal Ghazi Almalki, Ali Saleh Alsudais, Abdulrahman Sulaiman Alquzi, Mohamed Eldigire Ahmed, Mushtaq Ahmad Mir, Moudi M. Alasmari

**Affiliations:** ^1^Department of Pediatric, Ministry of National Guard Health Affairs (NGHA), King Abdulaziz Medical City, Jeddah, Saudi Arabia; ^2^Department of Basic Medical Sciences, College of Medicine, King Saud Bin Abdul Aziz University for Health Sciences (KSAU-HS), King Abdullah International Medical Research Center (KAIMRC), King Abdulaziz Medical City, Jeddah, Saudi Arabia; ^3^College of Science and Health Professions, King Saud Bin Abdul Aziz University for Health Sciences (KSAU-HS), King Abdullah International Medical Research Center (KAIMRC), Jeddah, Saudi Arabia; ^4^Department of Clinical Laboratory Science, College of Applied Medical Sciences, King Khalid University, Abha, Saudi Arabia

## Abstract

**Background:**

The prevalence of Clostridium difficile infection (CDI) as a common complication among inflammatory bowel disease (IBD) has been reported to increase worldwide and has been associated with a poor IBD outcome.

**Objectives:**

In this study, our aim was to report on the prevalence of CDI among IBD vs. non-IBD patients in King Abdulaziz Medical City (KAMC).

**Methods:**

This retrospective descriptive study was carried out between 2016 and 2020. Data of 89 patients reported with CDI in KAMC were analyzed for demographics and correlations between various characteristics such as BMI, personal/family history of IBD, infection with CDI, diagnosis, method of diagnosis, and treatment modalities.

**Results:**

Of the total 89 CDI patients, 59 (66.3%) were adults and 30 (33.7%) were pediatric, of which 36 (40.4%) were females and 53 (59.6%) were males. PCR was the main method of choice for the diagnosis of CDI (89.9%) followed by a positive-culture result (10.0%). Seventy-eight (87.6%) CDI patients were found to be immunocompromised, with two patients diagnosed with IBDs, one with UC, and one with CD. The recurrence rate was 38.4 (30 patients) among the immunocompromised group in comparison to 27.2 (3 patients) in the immunocompetent group (*p*=0.584).

**Conclusion:**

In this study, we found that adults were more prone to CDI infection, especially within hospital settings, and most of the CDI infections occurred in immunocompromised individuals, with cancer as the most common cause of it.

## 1. Introduction

In recent years, Clostridium difficile infection (CDI) has shown an increased incidence across the globe [1]. CDI is also considered one of the most common nosocomial infections bearing with it the risk of Clostridium difficile-associated diarrhea (CDAD) which can potentially be life-threatening [1]. Clostridium difficile (CD) is a Gram-positive spore-forming anaerobic bacillus that constitutes a part of the gut's normal flora in both humans and animals [1]. CDI is a contagious infection transmitted through the fecal-oral route in the form of spores that can be found in foods or any contaminated surfaces in an environment [2, 3]. In addition, asymptomatic carriers and infected patients are potential CDI reservoirs [4].

Deshpande et al. estimated that the incidence of CDI from the period of 2003–2009 has been increasing by 57% in pediatric patients; furthermore, out of 8,277,876 pediatric patients, 21,973 were diagnosed with CDI [5]. CDI clinical manifestations vary with the infection severity; however, symptoms primarily include diarrhea and other nonspecific symptoms such as fever, abdominal pain, and loss of appetite [5, 6]. The use of antibiotics is the standard of care for treating CDI patients. Another promising choice of treatment that could be used alternatively and has the highest rate of preventing recurrence is fecal microbiota transplantation, yet it raises several concerns, the chief being the possibility of pathogen transmission [1]. Nevertheless, some patients encounter a relapsed episode of CDI within 8 weeks after the previous episode. Almost one-third of CDI patients who are responsive to therapy develop recurrent episodes of CDI [6, 7].

CD spores, once within the host and soon after evading the gastric defenses, germinate into a toxin-releasing form that essentially drives the entire infection process. Spore germination decisively determines the occurrence of CDI especially in vulnerable individuals [3]. Prominent risk factors of CDI include old age, hospitalization, and administration of certain medications such as broad-spectrum antibiotics, proton pump inhibitors (PPIs), and histamine H2-receptor antagonists (H2RAs) [1, 2]. Antibiotics, PPIs, and H2RAs share the same outcome of disturbing the microbiota of the gut resulting in CDI [2, 5]. Penicillin, cephalosporin, clindamycin, and fluoroquinolone are among the broad-spectrum antibiotics that have been associated with CDI [5]. Surprisingly, prior use of vancomycin and metronidazole, which are used for CDI treatment, are also considered as key risk factors for CDI [1]. Populations aged 65 or older have an increased risk of CDI due to the presence of a high virulence strain of CD (BI/NAP1/027) and comorbidities such as diabetes mellitus (DM), tumors, and IBD [5, 7].

Inflammatory bowel disease (IBD) including Crohn's disease and ulcerative colitis are conditions in which prolonged chronic inflammation of the gastrointestinal (GI) tract results in irreversible impairments of the GI layers and functions [8]. These impairments can manifest as abdominal pain, rectal bleeding, persistent diarrhea, weight loss, and fatigue. Crohn's disease can result in damage to any part of the GI tract.

On the other hand, ulcerative colitis is only limited to the large intestine and the rectum [5, 8]. Statistically, the highest incidence of IBD among pediatric populations in Europe and Asia/the Middle East was estimated to be 23/100000 and 11.4/100000 person-years, respectively [9]. Although a definitive hypothesis about the etiology of this disease has not been established yet, researchers currently believe that genetic predisposition in addition to the exposure to environmental factors can lead to an alteration in the gut microbiota (dysbiosis) which triggers inflammation leading to IBD [10].

Hourigan et al. suggested that there were significant differences in the rate of CDI in both adult and pediatric populations with hospitalized IBD patients as high as 12 and 4 times, respectively [11]. Moreover, higher rates of recurrence, morbidity, and mortality as well as a more severe form of CDI have also been found in IBD patients [12]. A global study reported that the rate of recurrent CDI among hospitalized pediatric IBD patients was 34% contrary to 7.4% of nonhospitalized patients [11, 12]. In Saudi Arabia, there is a scarcity of research investigating recurrent CDI rates among the IBD population. Thus, this current study aims to estimate the prevalence of CDI among the IBD population in comparison with the population without IBD in King Abdulaziz Medical City in Jeddah. This study aims to estimate the prevalence of Clostridium difficile infection (CDI) among pediatric patients and adults with IBD in comparison to non-IBD patients at the National Guard Hospital in Jeddah, Saudi Arabia, with the following specific objectives:

1. Evaluate and compare the prevalence of CDI in both adult and pediatric patients
2. Assess the recurrence of CDI among IBD patients
3. Evaluate the response to treatment among CDI

## 2. Materials and Methods

### 2.1. Study Design and Setting

This study was a descriptive retrospective one carried out between the period of 2016 and 2020. The chart review of medical records from the patient care system (BESTCare) at National Guard Health Affairs in Jeddah, Saudi Arabia, was performed between the years of 2016 and 2020.

### 2.2. Consent and Ethical Approval

The study was carried out in line with the Helsinki protocol, and an ethical approval from the Institutional Review Board of King Abdullah International Medical Research Center (KAIMRC), KSAU-HS, Jeddah, was duly acquired before conducting this study. None of the names and IDs were collected from the participants, and the data were stored within 64-bit encrypted software on the Work PC of the PI, that was not prone to be breached by nonauthorized persons.

### 2.3. Study Participants and Sampling

The participants in this study were (a) all patients who were diagnosed with positive CDI, (b) of both genders, and (c) of age above 1 year. We excluded all immunocompromised patients. All patients were indiscriminately selected, and a convenience sampling method was used for the selection. The sample size was calculated by using the Raosoft® software (website link: https://www.raosoft.com/samplesize.html). The required sample size was calculated at the 90% confidence level with an estimated 50.0% prevalence of awareness regarding euthanasia and a margin of error of ±5%. A sample size of 128 was deemed fit as per the prevalence of the CDI in KAMC.

For the diagnosis of CDI, dual positive tests were regarded as the confirmation of CDI: (1) a stool culture on selective medium (TCCA: taurocholate cycloserine cefoxitin agar) and (2) a stool cytotoxicity assay on MRC-5 cells. However, for the cases in which a positive culture and negative stool cytotoxicity assay were obtained, a toxigenic culture (determination of the isolate's ability to produce toxins *in vitro*) was performed.

### 2.4. Data Collection Tools and Technique

A comprehensive review of the data/charts of the medical records from the patient care system (BEST Care) was conducted by a team of researchers for the extraction of data. The data extracted contained the information about the demographics, basal metabolic index (BMI), personal/family history of IBD, infection with CDI, diagnosis, method of diagnosis, and comorbidities. Treatment modalities of the patients were also reviewed and recorded in the data collection sheet.

### 2.5. Statistical Analysis

The data collected were tabulated, and analysis was performed using IBM SPSS Statistics for Windows, version 20.0. Descriptive analyses were conducted for frequencies and percentages, and mean values were obtained for continuous data. The chi-square (*χ*^2^) test was used to compare categorical variables in the questionnaire (gender, level of education, and college). *P* values less than 0.05 were accepted as statistically significant.

## 3. Results

### 3.1. Sociodemographic Characteristics

A total of 89 patients were diagnosed with CDI in this study, out of which 59 (66.3%) were adults and 30 (33.7%) were pediatric, and among them, 36 (40.4%) were females and 53 (59.6%) were males. The median age was 6.5 (6) and 6.3 (28) for pediatric and adult groups, respectively, with the median BMI of 23 (12.1%) for all groups combined (Table 1).

### 3.2. Diagnosis

PCR was the main method of choice for the diagnosis of CDI (89.9%) followed by a positive-culture result (10.0%). The overall recurrence rate was reported to be 37.1 (33 patients) (Table 2).

### 3.3. Correlations

**Figure 1:**
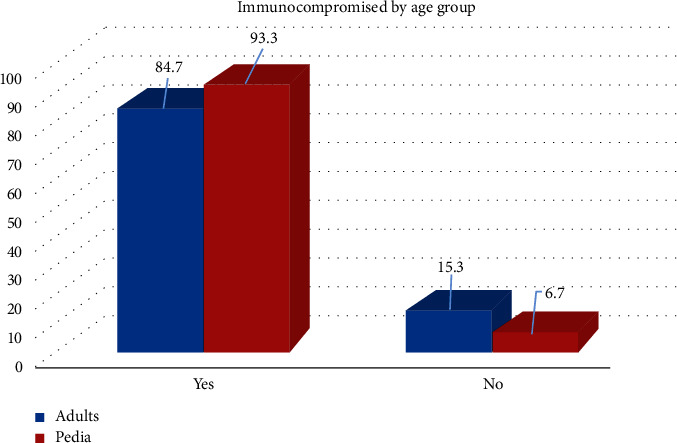
Immunocompromised by age group.

Seventy-eight (87.6%) CDI patients were found to be immunocompromised, with two patients diagnosed with IBDs, one with UC, and one with CD. Among those, 46 (58.9%) were males and 32 (41.1%) were females (*p*=0.768), and among them, 50 (64.1%) were adults and 28 (35.9%) were pediatrics (*p*=0.244). Furthermore, out of 78 immunocompromised patients, 69 (88.5%) were diagnosed with PCR and 9 (11.5%) were diagnosed with a positive-culture result (*p*=0.234) (Table 3, Figure 1).

**Figure 2:**
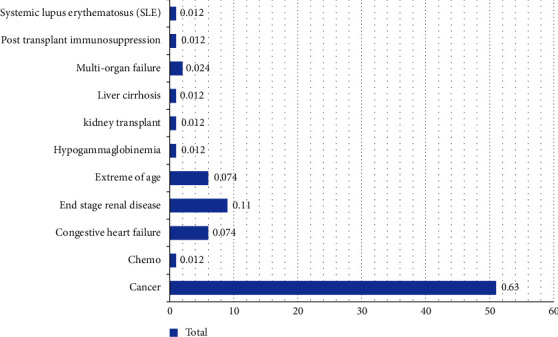
Etiology of immunosuppression in CDI-positive patients.

The recurrence rate was 38.4 (30 patients) among the immunocompromised group in comparison to 27.2 (3 patients) in the immunocompetent group (*p*=0.584). The 2 IBD cases were found to be immunocompetent (*p*=0.014). In addition, 84.7% of the adult patients were found to be immunocompromised. For the immunosuppression, cancer was by far the most common of the etiology (Figure 2).

## 4. Discussion

Clostridium difficile infection (CDI) is one the most common causes of nosocomial infection in developed countries and is lately emerging as the chief cause of morbidity and mortality in hospitalized patients [13–15]. Transient dysbiosis of the intestinal microbiota is typically regarded as the key risk factor for the primary and recurrent CDI; the other factors include hospitalization, antibiotic exposure, usage of proton pump inhibitors (PPIs), prior history of CDI, age more than 65, female gender, chemotherapy, immune suppression, and multiple comorbidities [16, 17].

IBD patients are at a higher risk of CDI infection due to dysbiosis and immunosuppression with ulcerative colitis (UC) patients having a higher risk in comparison to Crohn's disease (CD) [18, 19]. Among IBD patients, CDI infection is associated with poorer outcomes than those without CDI, for example, having longer hospital stays, higher rates of colectomies, and increased mortality [14, 15, 19, 20].

Furthermore, rates of CDI recurrences and colectomy have been observed to be higher in the IBD population than in the non-IBD population [18, 20]. In general, CDI is less commonly found in children; however, with an increase in the prevalence of pediatric IBD over the past 20 years, the burden of CDI is comparable among all age groups in IBD patients [21]. In addition, IBD children with CDI have more CDI recurrence rates and longer hospital stays compared to non-IBD ones [11, 22–24].

In our study, the highest incidence of CDI was seen in adults and not children. Although it is widely recognized that the relative risk of CDI is higher among young individuals [25], our results demonstrated that CDI was more prevalent in adult patients (59/78, 87.6%) and 84.8% of whom were immunocompromised as well. Furthermore, the majority of our patients had a prior exposure to antibiotics (69.7%) which is in line with the reported ones [26] and 84.7% of the adult patients were immunocompromised. Prior exposure to antibiotics underscores the antibiotic therapy efficacy and outcomes, especially in patients with immunosuppression [27].

Furthermore, community-acquired CDI is however not associated with severity as is in the hospital-acquired CDI [13, 14]. Recently, results from the multicenter phase II trials demonstrated that vancomycin was more effective than metronidazole for achieving symptomatic cure for CDI and also to prevent the recurrence [28, 29]; thus, for initial non-fulminant CDI, vancomycin or fidaxomicin are now recommended as the first line treatment [18]. In our study also, we found the recurrence of CDI in 33 (37.1%) of the patients who were treated with the first line of treatment.

## 5. Study Limitations

1. The sample population in this study was from a single center in Jeddah, and hence, the results do not necessarily reflect that of the general population
2. The study with its retrospective nature has an inherent inability to determine if there are regional variations in the incidence and prevalence of the disease

## 6. Conclusion

In our sentinel study, we found that adults are more prone to CDI infections within hospital settings. The highest incidence of CDI was seen among adults. In addition, most of the CDI infections occurred in the immunocompromised individuals. Among CDI-positive patients, cancer was found to be the most common cause for the immuocompromise. Further studies are warranted to analyze the association of various risk factors with the complications of CDI.

## Figures and Tables

**Table 1:** Basic characteristics of patients.

Variables	*N* = 89
Age (all)^*∗*^	48.0 (57.0)
Age (adults)^*∗*^	63.0 (28.0)
Age (pedia)^*∗*^	6.5 (6.0)

*Age group*	
Adult	59 (66.3)
Pediatric	30 (33.7)

*Gender*	
Female	36 (40.4)
Male	53 (59.6)
BMI (all)^*∗*^	23.0 (12.1)
BMI (adults)^*∗*^	26.5 (7.7)
BMI (pedia)^*∗*^	15.3 (3.0)

^*∗*^Median (IQR).

**Table 2:** Disease characteristics.

Variables	*N* = 89
*Immunocompromised*	
Yes	78 (87.6)
No	11 (12.4)

*Diagnosis*	
Crohn disease	1 (1.1)
Ulcerative colitis	1 (1.1)
No IBD	87 (97.8)

*Method of diagnosis*	
PCR	80 (89.9)
Culture	9 (10.1)
Prior exposure to antibiotics	62 (69.7%)
Treatment duration (all)^*∗*^	10.0 (7.0)
Treatment duration (adults)^*∗*^	10.0 (6.5)
Treatment duration (pedia)^*∗*^	10.0 (7.6)

*Response to treatment*	
Recurrent	33 (37.1)
Resolved	55 (61.8)
None	1 (1.1)

^*∗*^Median (IQR).

**Table 3:** Immunocompromised patient's characteristics.

Variables	Yes = 78	No = 11	*P* value
Age^*∗*^	50.5 (55.0)	27.0 (34.0)	0.645

*Age group*			
Adult	50 (84.7)	9 (15.2)	0.244
Pediatric	28 (93.3)	2 (6.7)	

*Gender*			
Female	32 (88.9)	4 (11.1)	0.768
Male	46 (86.8)	7 (13.2)	
BMI^*∗*^	23.6 (12.3)	22.3 (7.7)	0.556

*Diagnosis*			
Crohn disease	0 (0)	1 (100)	0.014
Ulcerative colitis	0 (0)	1 (100)	
No IBD	78 (89.7)	9 (10.3)	

*Method of diagnosis*			
PCR	69 (86.3)	11 (13.7)	0.234
Culture	9 (100)	0 (0)	
Treatment duration^*∗*^	10.0 (7.5)	10.0 (3.0)	0.930

*Response to treatment*			
Recurrent	30 (90.9)	3 (9.1)	0.584
Resolved	47 (85.5)	8 (14.5)	
None	1 (100)	0 (0)	

## Data Availability

The data used to support the findings of the study are available from the corresponding author upon request.

## Abbreviations

BMI: Basal metabolic index
CD: Clostridium difficile
CD: Crohn's disease
CDAD: Clostridium difficile-associated diarrhea
CDI: Clostridium difficile infection
DM: Diabetes mellitus
GI: Gastrointestinal
H2RAs: Histamine H2-receptor antagonists
IBD: Inflammatory bowel disease
KAIMRC: King Abdullah International Medical Research Center
KSAU-HS: King Saud Bin Abdulaziz University for Health Sciences
PPI: Proton pump inhibitors
TCCA: Taurocholate cycloserine cefoxitin agar
UC: Ulcerative colitis.
